# Genome-Wide Identification and Analysis of *Nilaparvata lugens* microRNAs during Challenge with the Entomopathogenic Fungus *Metarhizium anisopliae*

**DOI:** 10.3390/jof7040295

**Published:** 2021-04-14

**Authors:** Jiaqin Xie, Yifan Peng, Yuxian Xia

**Affiliations:** 1 Genetic Engineering Research Center, School of Life Sciences, Chongqing University, Chongqing 401331, China; pyifan@yeah.net; 2 Chongqing Engineering Research Center for Fungal Insecticides and Key Laboratory of Gene Function and Regulation Technology under Chongqing Municipal Education Commission, Chongqing 401331, China

**Keywords:** entomopathogenic fungus, *Metarhizium anisopliae*, fungal infection, *Nilaparvata lugens*, microRNAs, pest control

## Abstract

The resistance of the notorious rice pest *Nilaparvata lugens* to many insecticides has caused significant concerns. Our previous study demonstrated that the fungus *Metarhizium anisopliae* CQMa421 shows great potential for the control of this pest, but the interactions between them are still unclear. Thus, we further investigated fungal infection-related microRNAs (miRNAs) in *N. lugens* during *M. anisopliae* CQMa421 challenge using Illumina sequencing. In this study, we constructed twenty-four small RNA libraries over different time courses (i.e., 4 h, 8 h, 16 h, and 24 h). A total of 478.62 M clean reads were collected, with each sample producing more than 13.37 M reads, after the removal of low-quality reads. We identified 2324 miRNAs and their 11,076 target genes within the twenty-four libraries by bioinformatics analysis. Differentially expressed miRNAs (DEmiRNAs), including 58 (32 upregulated vs. 26 downregulated), 62 (30 upregulated vs. 32 downregulated), 126 (71 upregulated vs. 55 downregulated), and 109 (40 upregulated vs. 69 downregulated) DEmiRNAs were identified at 4 h, 8 h, 16 h, and 24 h post-infection, respectively. We further conducted Gene Ontology (GO) and Kyoto Encyclopedia of Genes and Genomes (KEGG) pathway analysis to predict the functions of all target genes of DEmiRNAs. These DEmiRNAs targets identified during 24 h of infection were primarily involved in energy metabolism, lysine degradation, the FoxO signaling pathway, ubiquitin-mediated proteolysis, the mRNA surveillance pathway, and the MAPK signaling pathway. Taken together, our results provide essential information for further study of the interactions between the entomopathogenic fungus *M. anisopliae* and *N. lugens* at the posttranscriptional level.

## 1. Introduction

The rice planthopper *Nilaparvata lugens* is one of the prominent insect pests associated with rice growth and production [1,2]. The area affected by rice planthoppers in China reached 23 million ha. in 2020 alone. This pest not only sucks the phloem sap of rice plants, causing direct damage [3], but also transmits plant viruses such as rice grassy stunt virus, resulting in indirect damage to rice plants [4]. Currently, the most common and effective approaches for the control of this pest depend on the use of chemical insecticides, whereas the misuse of insecticides has contributed undesirable effects on the ecological environment and nontargets as well as human health [5,6]. This species also rapidly develops resistance, adapting to insecticides after several generations, thus resulting in resurgence [1,7]. Transgenic rice varieties and approaches such as RNAi have been developed for insect pest control and achieved some success, but these methods are limited in terms of their effectiveness and availability for field applications [8,9,10]. Thus, more effective alternative approaches are needed to replace/reduce the use of chemical insecticides for pest control, especially under field conditions.

Entomopathogenic fungi are one of most important factors regulating the insect populations under natural conditions [11,12]. In contrast to the side effects of chemical insecticides, insects do not easily become resistant to fungal infection, which also poses lower risks to the environment and human health [11]. Several entomopathogenic fungal strains have been isolated from natural hosts and used for the control of some insect pests. For instance, two important fungal agents, *Metarhizium anisopliae* and *Beauveria bassiana*, showed good potential against insect pests such as *Locusta migratoria* [13], *Alphitobius diaperinus* [14], *Chironomus riparius* [15], and *Helicoverpa armigera* [16]. Additionally, a few specific *M. anisopliae* strains can be used against insecticide-resistant pests [17]. The engineered fungal strains transformed with genes encoding products such as an insecticidal scorpion toxin (Bjα IT) and *Manduca sexta* diuretic hormone (MSDH) showed improved virulence for the target pests [18,19]. The combined use of *M. anisopliae* and RNAi can affect the reproduction of *N. lugens* and has shown good potential for the control of this pest [20]. In our previous study, we found that the fungus *M. anisopliae* CQMa421 exhibited great potential for the control of major rice pests, including the rice planthopper *N. lugens* [21]. This fungal agent can also suppress the population of rice planthoppers to a low level under field conditions [22,23]. However, the underlying molecular interactions between the rice planthopper and the fungus are poorly understood, although this knowledge may enhance potential fungal agents for other pest control.

MicroRNAs (miRNAs) are a class of small noncoding RNAs consisting of ~22 nucleotides, including small interfering RNAs (siRNAs) and piwi-interacting RNAs (piRNAs) [24,25]. These miRNAs are common in plants, insects, and microbes and play crucial roles in regulating gene expression at the posttranscriptional level [26,27,28]. Many studies have reported that miRNAs can affect diverse physiological processes in organisms, such as development [29], metamorphosis [30,31], sexual divergence [32], and wing polyphenism development [33,34]. On the other hand, the uncontrolled expression of miRNAs may result in undesirable results, such as disease, unusual phenotype variation, or death [35]. With the ongoing development of sequencing technologies, miRNAs have been evaluated in many insect species under different conditions, including responses to stressful treatments or pathogenic challenges, which have provided important information for investigating the roles of miRNAs in regulating their target mRNAs [36,37,38]. These data also facilitate the development of new approaches for pest control in terms of providing potential target genes for both RNAi technology and the development of engineered fungus.

In a previous study, we evaluated the potential of *M. anisopliae* to infect the rice pest *N. lugens* adults and nymphs [21]. A large scale application of this fungal agent may also suppress the pest populations under field conditions, indicating that it can be employed as an alternative to chemical insecticides [23]. Although a few studies have reported the mRNA responses of such pests to different challenges, including insecticides, temperature stress, or different developmental conditions, the levels of fungus-induced miRNAs in these pests have scarcely been studied [39,40,41]. However, the study of miRNAs is crucial to reveal the interactions between the fungus and the insect host at the posttranscriptional level. Thus, we further examined the resulting miRNAs of *N. lugens* in response to *M. anisopliae* infection during different periods by transcriptomic analysis. This investigation may provide new insights for further study of insect host responses to entomopathogens at the posttranscriptional level and understand the molecular mechanisms of fungal infection.

## 2. Methods and Materials

### 2.1. Insect and Sample Collection

The *N. lugens* insects used in this study were obtained from our insectary (Plant Experimental Base at Chongqing University, Chongqing, China). The population of *N. lugens* was maintained on fresh rice seedlings under the following conditions: 27 ± 1 °C with a light:dark (L:D) photoperiod of 14:10 h. To acquire the experimental insect samples, we randomly collected the *N. lugens* nymphs and then treated them with the fungal *M. anisopliae* CQMa421. The preparation of a suspension of 1 × 10^8^ conidia/mL of *M. anisopliae* was performed as described in our previous study [21].

For individual insect treatment, we first transferred 10 fresh rice seedlings to a column bucket with a diameter of 10 mm and a height of 150 mm. Then, nymphs of *N. lugens* were inoculated on the rice plants and sprayed with the prepared *M. anisopliae* suspension. After this treatment, the *N. lugens* nymphs were incubated in a bioassay room under a 27 ± 1 °C and 14:10 h (L:D) photoperiod. The control-group individuals were treated with distilled water using the same method indicated above. Finally, *N. lugens* nymphs from both the *M. anisopliae*-treated and control groups were collected after incubation on the rice seedings for 4 h, 8 h, 16 h, or 24 h. Three replicates were performed for each group, and a total of twenty-four sample groups of *N. lugens* individuals were flash frozen in liquid nitrogen and stored prior to RNA extraction.

### 2.2. Small RNA (sRNA) Extraction and Sequencing

The whole bodies of *N. lugens* nymphs from the *M. anisopliae*-infected or noninfected groups were collected at 4 h, 8 h, 16 h, and 24 h posttreatment for sRNA sequencing. The total RNA of *N. lugens* nymphs was extracted using TRIzol reagent (Invitrogen, Carlsbad, CA, USA) according to the manufacturer’s instructions. The purity, concentration, and integrity of the RNA samples were tested using a NanoPhotometer^®^ spectrophotometer (IMPLEN, Westlake Village, CA, USA). The samples were evaluated in agarose gels to ensure that they showed a sufficiently high quality for transcriptome sequencing. The preparation of the sRNA sequencing library was performed as follows: first, the 3′ SR and 5′ SR adaptors were ligated to the ends of the sRNAs by using the T4 RNA ligase. Then, first-strand cDNA was synthesized by reverse transcription. PCR amplification was performed, and size selection was carried out via polyacrylamide gel electrophoresis (PAGE). The PAGE was used for fragment screening purposes, with rubber cutting recycling of the pieces to produce small RNA libraries. The PCR products were purified (AMPure XP system), and library quality was assessed. The clustering of the index-coded samples was performed on a cBot Cluster Generation System using the TruSeq PE Cluster Kit v4-cBot-HS (Illumina, San Diego, CA, USA) according to the manufacturer’s instructions. After cluster generation, the library preparations were sequenced on an Illumina platform, and single-end reads were generated by a service provider (BioMarker Technologies, Beijing, China). The Illumina sequence reads of *N. lugens* were deposited in the NCBI SRA database (accession no. PRJNA686491).

### 2.3. N. lugens miRNA Prediction

The raw reads in FASTQ format were first processed with in-house Perl scripts. In this step, clean reads were obtained by removing reads containing adapters, reads containing ploy-N sequences and low-quality reads for the raw data. In this treatment, all reads were trimmed and cleaned by removing sequences smaller than 18 nt or longer than 30 nt. Additionally, the Q20 and Q30 values, GC contents, and sequence duplication levels were calculated from the clean data. All downstream analyses were based on the clean data with high quality. Clean reads were obtained using Bowtie tools software via sequence alignment against the Silva database, GtRNAdb database, Rfam database, and Repbase database, and ribosomal RNAs (rRNAs), transfer RNAs (tRNAs), small nuclear RNAs (snRNAs), small nucleolar RNAs (snoRNAs), and other types of noncoding RNAs (ncRNAs) and repeats were filtered to identify and predict miRNAs. The remaining reads were used to identify known miRNAs and novel miRNAs by mapping with the *N. lugens* genome (NCBI project accession: PRJNA177647) [42] using the Bowtie program [43].

### 2.4. Differential Expression Analysis and Functional Annotation

For the differential expression analysis of the *M. anisopliae*-infected or noninfected *N. lugens* groups, we used the DESeq2 R package. DESeq2 provides statistical routines for determining differential expression from digital miRNA expression data by using a model based on the negative binomial distribution. The resulting P values were adjusted using the Benjamini and Hochberg approach for controlling the false discovery rate. All of the miRNAs with a |log_2_(FC)| ≥ 1.5 and *p* ≤ 0.05 predicted by DESeq2 were assigned as differentially expressed miRNAs (DEmiRNAs).

Prediction of the potential target genes of differentially expressed miRNAs was conducted using the miRanda and RNAhybrid software packages [44,45]. Gene functional annotation of *N. lugens* was based on the following databases: Nr (NCBI non-redundant protein sequences); Nr (NCBI non-redundant nucleotide sequences); Pfam (Protein family); Swiss-Prot (A manually annotated and reviewed protein sequence database); KO (KEGG Orthologue database); and GO (Gene Ontology). The expression of miRNAs in all libraries was normalized based on the TPM algorithm, and the formula was as follows:

TPM = Readcount × 10^6^/MappedReads

The genome database of *N. lugens* (*Nilaparvata lugens* NilLug1.0) used as the background to determine GO and KEGG terms enriched within the predicted targets dataset. Gene Ontology (GO) (http://www.geneontology.org/, accessed on 22 November 2019) enrichment analysis of the DEmiRNAs was implemented by using the GOseq R package-based Wallenius noncentral hypergeometric distribution to identify the significantly enriched terms [46]. Kyoto Encyclopedia of Genes and Genomes (KEGG) pathway enrichment analysis was conducted to predict and identify the significant pathways, with datasets generated by genome sequencing or other high-throughput experimental technologies (http://www.genome.jp/kegg/, accessed on 22 November 2019). KO-based Annotation System (KOBAS) is an open-access system to use KO as a controlled vocabulary to automatically annotate a set of sequences, which can identify the most frequent and the most significantly enriched pathways in a given set of sequences [47]. We used it to test the statistical enrichment of differential expression genes in KEGG pathways.

### 2.5. Validation by RT-qPCR

We used real-time quantitative PCR (RT-qPCR) to analyze the expression of genes and to confirm the results of RNA sequencing. The highly expressed 10 miRNAs among the DEmiRNAs were randomly selected. The RT-qPCR was performed on a Bio-Rad iQ2 optical system (Bio-Rad, Hercules, CA, USA) with a QuantiNove SYBR Green PCR Kit (QIAGEN, Dusseldorf, Germany) following the instructions of the manufacturer. The β-actin was used as an internal control. Each experiment was repeated in triplicate. Finally, data analysis was performed using the 2^−∆∆Ct^ method. The primers designed for RT-qPCR in this study are listed in Table S1.

## 3. Results

### 3.1. Overall and Size Distribution of N. lugens Total miRNAs

To identify the *N. lugens* miRNAs, we constructed 24 libraries after challenge with *M. anisopliae* for 4 h, 8 h, 16 h, and 24 h challenge by next-generation sequencing. The libraries generated for the control group and the *M. anisopliae*-infected group at different times were designated T-4 h vs. W-4 h, T-8 h vs. W-8 h, T-16 h vs. W-16 h, and T-24 h vs. W-24 h, respectively. In this analysis, a total of 541.47 M raw reads were generated, and 478.62 M clean reads were retained after removing the low-quality reads. The Q30 of all samples was greater than 94.19%, indicating good sequencing data quality without low-quality sequences (Table S2). The mapped reads of all samples, except for T8-1 (59.08%) and W4-3 (59.18%), were more than 60% (Table S3).

**Figure 1:**
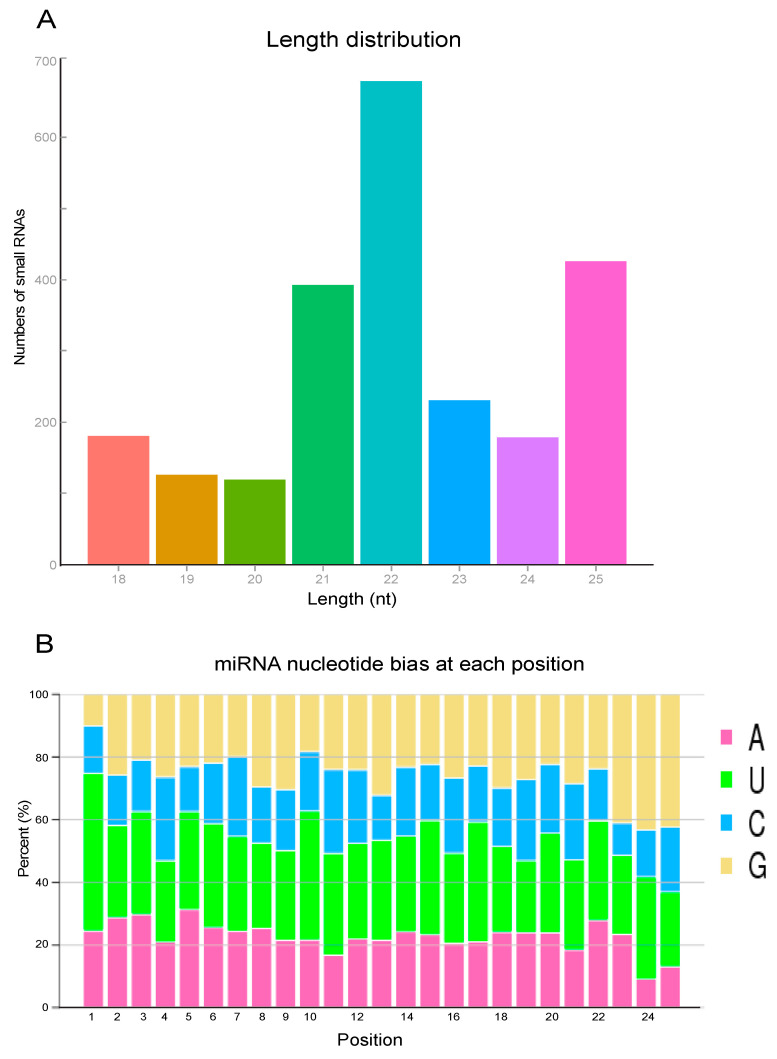
The length distribution and nucleotide bias of unique small RNA reads in the libraries of *N. lugens*. (**A**) The total length distribution of *N. lugens* small RNAs in the twenty-four libraries (4 h, 8 h, 16 h, and 24 h) generated after *M. anisopliae* infection; (**B**) the first nucleotide bias at each position among miRNAs of different lengths in the twenty-four libraries.

We noted that the peak of the total miRNA read length distribution occurred at 22 nt, representing the typical lengths of conserved miRNAs; and next most abundant lengths were 25 nt, corresponding to piRNA-like sequences (Figure 1A). Additionally, the examination of the first nucleotide bias of the miRNAs showed a strong preference for uracil “U” and adenine “A” (Figure 1B). The 5′ terminus of miRNA residues from 2 to 8 is believed to recognize the target mRNAs and repress posttranscription. The most abundant bases in such residues were U, U, U, A, U, U, and guanine “G”, respectively (Figure 1B).

### 3.2. DEmiRNA Analysis of N. lugens after Fungal Treatment

**Figure 2:**
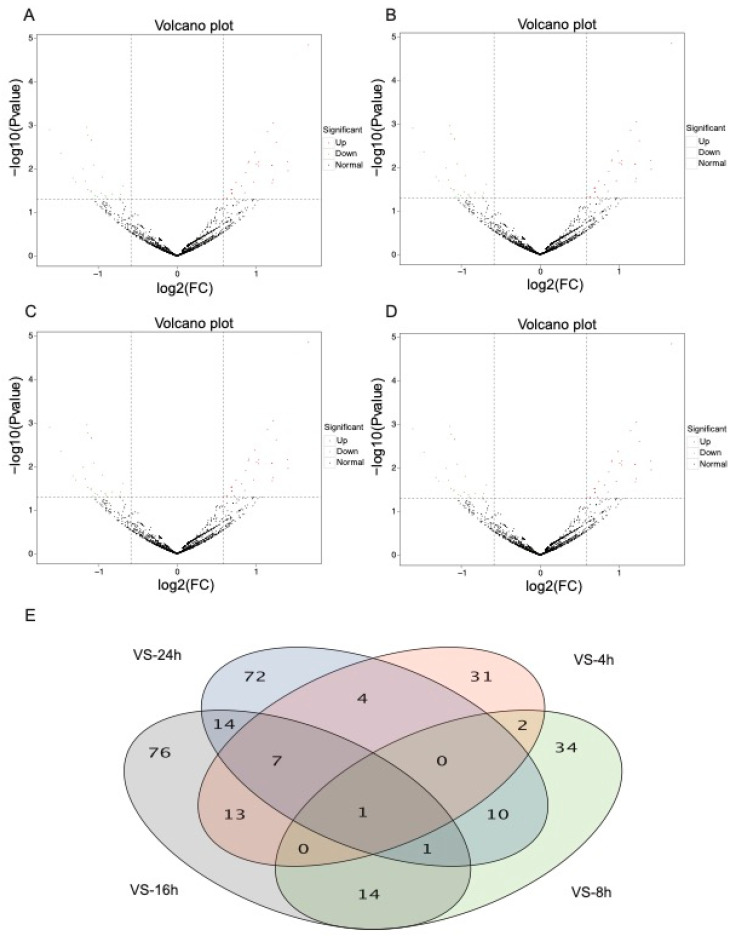
The volcano plot and numbers of differentially expressed miRNAs (DEmiRNAs) identified after *M. anisopliae* infection. (**A**) The volcano plot of DEmiRNAs at 4 h post-challenge; (**B**) the volcano plot of DEmiRNAs at 8 h post challenge; (**C**) the volcano plot of DEmiRNAs at 16 h post-challenge; (**D**) the volcano plot of DEmiRNAs at 24 h post-challenge; and (**E**) the Venn diagram of DEmiRNAs after *M. anisopliae* infection at 4, 8, 16, and 24 h.

To identify *N. lugens* DEmiRNAs after *M. anisopliae* challenge, we calculated the read counts for each of the miRNAs and compared their expression levels during the time-course of infection using DESeq2 according to the criteria of a *p* value < 0.05 and FC > 1.5. After filtration, a total of 355 DEmiRNAs were identified between all libraries from the infected and uninfected groups with the 24 h experiments. Specifically, 58 DEmiRNAs were identified after 4 h of infection, including 32 upregulated and 26 downregulated miRNAs (Figure 2A). After 8 h of fungal infection, 62 DEmiRNAs were identified, including 30 upregulated vs. 32 downregulated miRNAs (Figure 2B). We identified 126 DEmiRNAs at 16 h, including 71 upregulated and 55 downregulated miRNAs (Figure 2C). Finally, 109 DEmiRNAs were identified after 24 h of infection, including 40 upregulated and 69 downregulated miRNAs (Figure 2D). Only 1 DEmiRNA was identified in all four time-course treatment groups. Moreover, we found that the number of DEmiRNAs was variable at different times, showing an increasing tendency over time after fungal infection. The top ten *N. lugens* DEmiRNAs (up- or downregulated) after *M. anisopliae* infection also showed significant differences over the time course (Table S4).

### 3.3. Functional Analysis of DEmiRNAs in N. lugens

**Figure 3:**
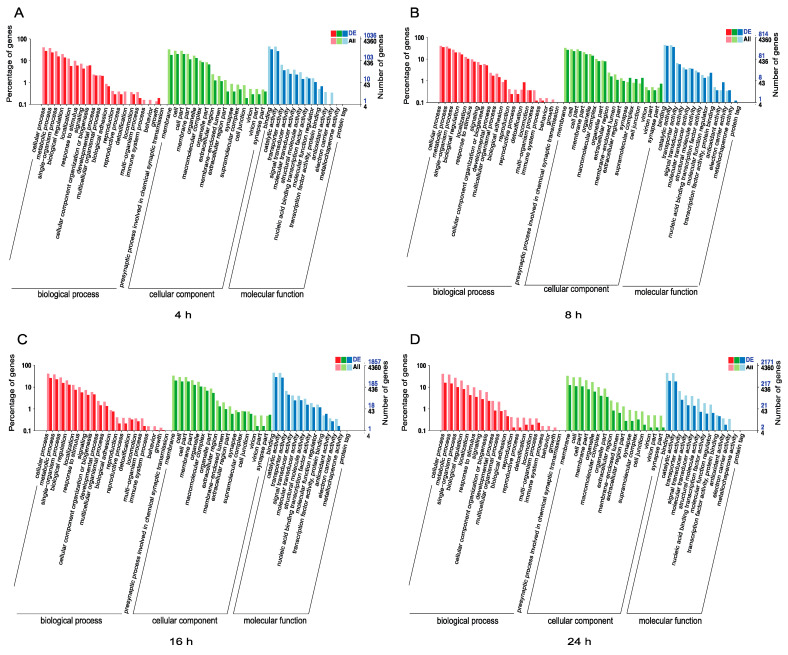
The GO categories of *N. lugens* DEmiRNAs identified during *M. anisopliae* infection. (**A**) The GO categories of *N. lugens* DEmiRNAs at 4 h post *M. anisopliae* infection; (**B**) the GO categories of *N. lugens* DEmiRNAs at 8 h post *M. anisopliae* infection; (**C**) the GO categories of *N. lugens* DEmiRNAs at 16 h post *M. anisopliae* infection, and (**D**) the GO categories of *N. lugens* DEmiRNAs at 24 h post *M. anisopliae* infection.

To further examine the functions of all DEmiRNAs in *N. lugens*, we predicted the target genes of the miRNAs in the GO databases and mapped them to different functions (Table S5). The annotated GO terms of the 4360 target mRNAs for the total DEmiRNAs predicated using RNAhybrid and miRanda algorithms were divided into the biological process, cell component, and molecular function categories. The differences in the abundances and numbers of the mRNAs associated with the four examined infection periods are shown according to the different GO categories of the 1036, 814, 1867, and 2171 target mRNAs identified after 4 h, 8 h, 16 h, and 24 h of infection, respectively. In the biological process category, the target genes of miRNAs were majorly associated with the in cellular process and metabolic process functions. Membrane and cell components were the most enriched cellular component categories, while binding and catalytic activity were the most enriched molecular functions. Interestingly, there were no target mRNAs associated with detoxification functions identified at 4 h (Figure 3A), while this functional category showed enrichment at 8 h, 16 h, and 24 h after infection (Figure 3B–D). Additionally, no target mRNAs were associated with the functions of antioxidant activity and electron carrier activity after 4 h of infection, but these two metabolic activity categories showed enrichment at 8 h and 16 h post-infection (Figure 3B,C).

**Figure 4:**
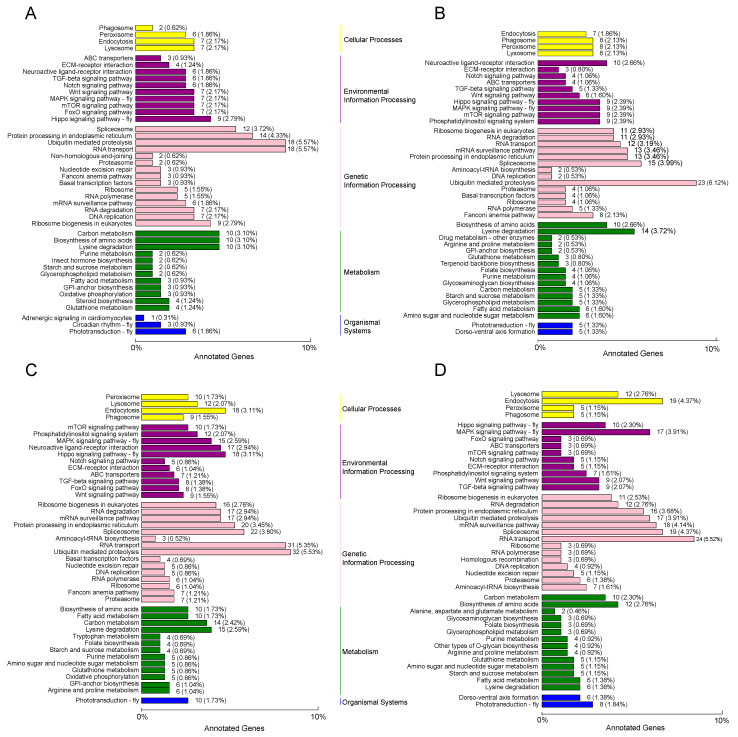
The enrichment and dispersion of differentially expressed miRNAs in KEGG pathways after *M. anisopliae* infection. (**A**) The identified pathways of DEmiRNAs after 4 h of *M. anisopliae* infection; (**B**) the identified pathways of DEmiRNAs after 8 h of *M. anisopliae* infection; (**C**) the identified pathways of DEmiRNAs after 16 h of *M. anisopliae* infection; and (**D**) the identified pathways of DEmiRNAs after 24 h of *M. anisopliae* infection. The asterisks indicate significant differences according to a *p* value < 0.05.

We further used KEGG to identify the metabolic and signal transduction pathways associated with the targets of the DEmiRNAs after *M. anisopliae* infection (Table S6). The target genes of 323 differentially expressed mRNAs identified after 4 h infection were mapped to 77 pathways in the KEGG database, including the FoxO signaling pathway, notch signaling pathway, steroid biosynthesis, etc. (Figure 4A). When *N. lugens* was challenged by *M. anisopliae* for 8 h, 82 pathways (including lysine degradation, ubiquitin mediated proteolysis, mTOR signaling pathway, etc.) were found to be enriched with the targeted genes of 376 differentially expressed mRNAs (Figure 4B). A total of 2166 targets of 579 differentially expressed mRNAs identified after 16 h infection was mapped to 93 pathways in the KEGG database (including the ubiquitin-mediated pathway, phototransduction, mRNA surveillance pathway, etc.) (Figure 4C). We found that there were 2085 genes targeted including 435 differentially expressed mRNAs identified after 24 h infection mapped to 89 pathways, including the MAPK signaling pathway, starch and sucrose metabolism, endocytosis, etc. (Figure 4D). The top KEGG targets of the DEmiRNAs are shown in Figure 4 and mainly included the lysine degradation (KO: 00310), ubiquitin-mediated proteolysis (KO: 04120), starch and sucrose metabolism (KO: 00500), RNA transport (KO: 03013) and FoxO signaling (KO: 04068) pathways.

From the highly expressed DEmiRNAs, the Nr annotation of the target DEGs included the serine/threonine-protein kinase mTOR (NL-miR-2333), serine/threonine-protein kinase PAK 3, insulin receptor, and E3 ubiquitin-protein ligase UBR2 (NL-miR-1047); thioredoxin domain-containing protein (NL-miR-156); and E3 ubiquitin-protein ligase MYCBP2 (NL-miR-488) at 4 h infection. Eight hours after *M. anisopliae* infection, the target DEGs mainly included the apoptosis-resistant E3 ubiquitin protein ligase 1, nucleoprotein TPR, diacylglycerol kinase theta isoform X3, and protein phosphatase PHLPP (NL-miR-260); cytosolic carboxypeptidase (NL-miR-1675); transient receptor potential channel pyrexia (NL-miR-2180); phospholipid phosphatase and zinc finger protein (NL-miR-707); glucose transporter (NL-miR-1385); and DNA topoisomerase (NL-miR-1547). The DEGs at 16 h included a serine/threonine-protein kinase, histone acetyltransferase, E3 ubiquitin-protein ligase, and extracellular sulfatase corresponding to NL-miR-1982; putative mediator of RNA polymerase II, GTPase-activating protein, phenoloxidase, and odorant receptor corresponding to NL-miR-1147; phospholipid-transporting ATPase corresponding to NL-miR-453; and serine/threonine-protein kinase and GTPase-activating protein corresponding to NL-miR-1462. We noted that the DEGs at 24 h included the heparin sulfate glucosamine, tRNA modification GTPase, histone-lysine N-methyltransferase, and glucose-6-phosphate 1-dehydrogenase (NL-miR-54); transcriptional regulator and histone acetyltransferase (NL-miR-1546); and activating signal co-integrator, tripeptidyl-peptidase, and pyruvate carboxylase (NL-miR-756) (Table S7).

### 3.4. Validation of DEmiRNAs by RT-qPCR

**Figure 5:**
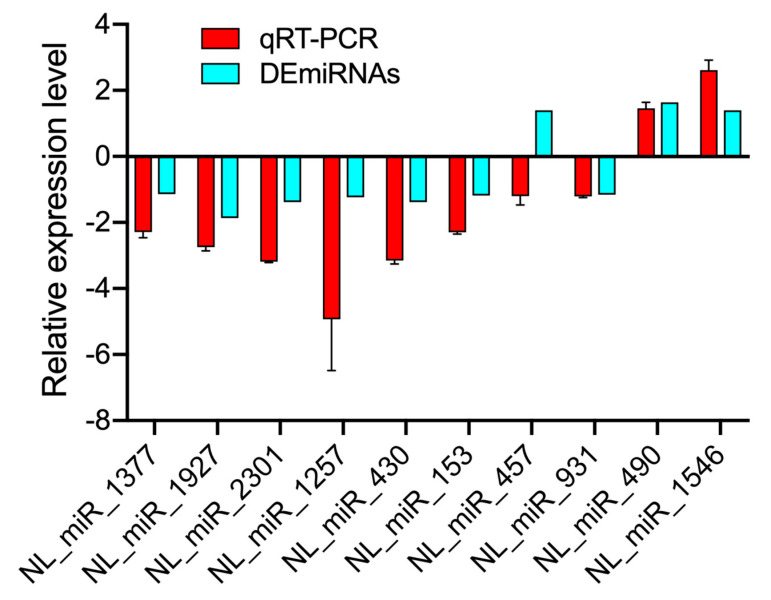
The validation of RNA sequencing and RT-qPCR results for target genes. Three replicates were performed for each miRNA, and error bars represent the mean ± SE.

To further validate the DEmiRNAs identified through sequencing, we used RT-qPCR to analyze gene expression and confirm the results of RNA sequencing. We selected 10 of the highly expressed miRNAs based on the Illumina sequencing results. The results showed that the expression trends of the selected miRNAs showed a slight discrepancy from the findings of the sequencing analysis (Figure 5), which might be due to the differences in the sensitivity, specificity, and applied algorithms between the two techniques.

## 4. Discussion

The field population of the pest *N. lugens* has evolved high resistance (more than 1000-fold) to many common chemical insecticides, such as imidacloprid and buprofezin [48]. The use of chemicals imposes significant negative effects on species diversity and the well-being of ecosystems [5,49]. Alternative control tools, such as entomopathogenic fungi, present good potential for the insect pest control under both greenhouse and large-scale field conditions [23,50]. The entomopathogenic fungus *M. anisopliae* has been used for the control of several insect pests, including the major rice pests. In our previous study, we isolated the fungal *M. anisopliae* strain CQMa421 to challenge *N. lugens* nymphs and adults, which resulted in high mortality. Additionally, this fungus has been suggested for use in long-term control under field conditions and has relatively limited effects on nontargets species (i.e., microbial diversity and structure) [22]. Although a few studies focused on the transcriptomic level of *N. lugens* under stressful conditions, less attention has been paid to the analysis of this fungus at the posttranscriptional level, especially under entomopathogenic fungal challenge. In this study, we compared the miRNAs of *N. lugens* after challenge with the entomopathogenic fungus *M. anisopliae* in different infection stages. This investigation at the posttranscriptional level will provide important insights for further study of the interactions between the entomopathogenic fungus and the insect host. These results may also contribute to the development of new strategies (i.e., new targets for RNAi) for the control of this pest.

*N. lugens* miRNAs were identified after the fungal *M. anisopliae* infection for 4 h to 24 h, and the results showed variation in terms of both the numbers and targets of the identified miRNAs. The targets of the miRNAs identified after *M. anisopliae* infection are involved in the many metabolic processes and pathways, including carbon metabolism, starch and sucrose metabolism, ubiquitin-mediated proteolysis, the FoxO signaling pathway, and the pentose phosphate pathway. To defend against pathogens, insect hosts have evolved complex mechanisms for responding to and eliminating pathogen infection [51]. The cuticle is a protective barrier that helps to recognize and defend against pathogenic infection [52]. When pathogens adhere to the insect cuticle, the pattern recognition molecules (i.e., peptidoglycan recognition proteins, β-1,3-glucan recognition proteins, scavenger receptors, and galectins) of the insect host are activated [53,54]. Moreover, the innate immune system, including cellular and humoral immune responses, of insect hosts plays important roles in defending against pathogens, although insects lack an adaptive immune response [55,56].

We found that a few miRNAs were specifically expressed under *M. anisopliae* infection at different times during initial post-infection, indicating that these miRNAs play vital roles in defending *N. lugens* against the entomopathogenic fungus *M. anisopliae.* Conidial adhesion, detoxification, and penetration of entomopathogenic fungi on the cuticle of insect hosts are important processes in initial infection [52]. During this period, certain proteins (MAD1, G-protein-coupled receptors (GPCRs), dehydrogenases, and lipases) and pathways (mitogen-activated protein kinase (MAPK) and protein kinase A (PKA) pathways) are involved in in these processes [57]. Other studies have shown that fungal infection may result in the expression of specific genes with different time courses [51]. Additionally, the number and type of hemocytes, which play key roles in cellular immunity, have been shown to vary with post-infection time [58], while our study did not check these changes.

In our study, there was no apparent immune response at 4 h post-*M. anisopliae* infection (Figure 3A), but we noted an immune response at 8 h post-infection. Furthermore, the targets of the detoxification process were first identified at 8 h post-infection and showed a high expression. After 8 h of fungal infection, miRNA targets with catalytic activity showed high expression, while chemical synaptic transmission was significantly inhibited at this time. We also noted that DEmiRNAs were slightly more abundant at 16 h than at 24h, and the target genes and pathways identified at these time points were different (Tables S4 and S7). While miR-197 showed high expression at 16 h, miR-277 and miR-237 exhibited low expression. In contrast, miR-1834 showed high expression at 24 h, when miR-165 showed low expression. According to these target mRNAs, an RNAi-based technology can be developed and further combined with fungal agents for the control of insect pests. In fact, a previous study has been conducted to evaluate their combined use for rice pest control, suggesting a good potential for this pest control [20].

The most common metabolic processes and pathways identified within the initial 24 h after *M. anisopliae* infection were recognition, energy metabolism, the FoxO signaling pathway, and the MAPK signaling pathway, indicating these physiological responses in *N. lugens* are intense during fungal infection. These pathways are important for regulating organismal development, immune responses, and behavior [59,60]. In this study, 58 of the DEmiRNAs at 4 h targeted DNA replication, lysine degradation, ubiquitin-mediated proteolysis, and RNA transport. After 8 h, the top pathways of the targets were related to lysine degradation, ubiquitin-mediated proteolysis, starch and sucrose metabolism, and the mRNA surveillance pathway. We noted that there were specific pathways identified at 16 h (i.e., the Hippo signaling pathway) and 24 h (i.e., the MAPK signaling pathway) after *M. anisopliae* infection. The identified DEmiRNAs and targeting pathways exhibited differences in abundance during *M. anisopliae* infection from 4 to 24 h. In fact, several studies have shown that different developmental stages and sexes also exhibit miRNA differences [30,32]. The temporal patterns between host and fungus have also been studied in the insect *Plutella xylostella*, and the results showed time-dependent expression [61]. Furthermore, we noted that the qPCR result showed a slight discrepancy from the sequencing results, and we believe that a time-point selection of miRNAs for qPCR would better reflect the sequencing results.

In conclusion, we identified and analyzed the posttranscriptional regulation of miRNAs after *M. anisopliae* infection. A total of 2324 miRNAs, including 355 DEmiRNAs, were identified and found to target 11,076 genes (Table S5). Our results revealed the interactions between the *N. lugens* host and the infecting entomopathogenic fungus *M. anisopliae* and provide important insights for further research into the role of *N. lugens* miRNAs in responding to fungal infection. They may also contribute to the development of control strategies for this pest.

## Data Availability

All sequence data are available in the NCBI GenBank following the accession numbers (PRJNA686491) in the manuscript.

## Supplementary Materials

The following are available online at https://www.mdpi.com/article/10.3390/jof7040295/s1, Table S1: Primers used for RT-qPCR in this study, Table S2: Statistical summary of *N. lugens* miRNAs identified in the *M. anisopliae*-infected and noninfected groups. Table S3: The information of mapped reads of all samples after *M. anisopliae* treatment or control group. Table S4: Top ten up- and down-regulated DEmiRNAs in *N. lugens* after *M. anisopliae* challenge. Table S5: The number information of annotated targets of miRNAs with different database. Table S6: The identification of pathways, target genes of *N. lugens* miRNAs after fungal infection during different times. Table S7: Predicted targets of highly expressed DEmiRNAs in *N. lugens*.
