# Supplementary material for: Genome-Wide Identification and Analysis of Nilaparvata lugens microRNAs during Challenge with the Entomopathogenic Fungus Metarhizium anisopliae
Source: J Fungi (Basel). 2021 Apr 14;7(4):295. doi: 10.3390/jof7040295 (PMC8070897; doi:10.3390/jof7040295)
Supplement: Supplementary file 1 [file jof-07-00295-s001.pdf]

Table S1. Primers used for RT-qPCR in this study.

Gene ID	Primer sequences
NL-miR_1377	CGCGCGTTTTGTGCTCTGTGTTAT
NL-miR_931	TGGGTTGACAATGGGCAATGAAGC
NL-miR_430	TTCCATTCCATTGCAAGCTGCACC
NL-miR_2301	CGCAATCTCGTTCCGACCAGATTTTT
NL-miR_1927	CCTTACTTTCTTGAGATGGTGCCAGGT
NL-miR_153	GCGCGATCAGACTGTGACAAACT
NL-miR_1257	CGCGCCTGTAATGGACCAAACTTTT
NL-miR_490	CCGCGTAGTTAGAAGTATCGAAAAGGGT
NL-miR_457	CGCGTGGAACTGGTTCTGGAA
NL-miR_1546	CGCGTGGAACTGGTTCTGGAA
N.L-β-actin_F	CCAACCGTGAGAAGATGACC
N.L-β-actin_R	GATGTCACGCACGATT

Table S2. Statistical summary of *N. lugens* miRNAs identified in the *M. anisopliae*-infected and noninfected groups.

No.	Samples	Raw reads	Length<18	Length>30	Clean reads	Q30(%)	Low quality
1	T4-1	28336440	537776	2635662	25162991	97.73	0
2	T4-2	26171434	526141	2558487	23086793	97.86	0
3	T4-3	28826809	432510	1765362	26628928	97.76	0
4	T8-1	15665208	1404151	622465	13637861	96.51	0
5	T8-2	18468911	370356	1498074	16599640	96.52	0
6	T8-3	22478979	449182	1399148	20629683	96.49	0
7	T16-1	22456925	429857	2053780	19973277	97.77	0
8	T16-2	27262922	400525	3162880	23699498	97.68	0
9	T16-3	27327380	1630564	2540885	23155920	97.85	0
10	T24-1	24542904	1329713	1734788	21478403	97.83	0
11	T24-2	22488989	1286903	1869205	19332881	97.64	0
12	T24-3	22505235	1097442	1393644	20014149	97.86	0
13	W4-1	17747182	59004	2259231	15428946	94.97	0
14	W4-2	14895784	45151	1477341	13373290	94.7	0
15	W4-3	16677157	119831	2850898	13706427	94.92	0
16	W8-1	23581508	120973	3223812	20236722	94.97	0
17	W8-2	17848523	105520	1727071	16015932	94.81	0
18	W8-3	19232214	1039381	1206220	16986612	94.19	0
19	W16-1	19478228	343663	1620975	17513589	94.98	0
20	W16-2	17709816	283370	1533452	15892993	95.19	0
21	W16-3	17209078	689948	1208656	15310473	95.05	0
22	W24-1	34508602	971277	2740808	30796504	97.63	0
23	W24-2	26643095	1138505	1957434	23547151	97.63	0
24	W24-3	29406432	1298075	1700929	26407410	97.84	0

T, the fungal *M. anisopliae*-infected treatment; W, Control group.

Table S3. The information of mapped reads of all samples after *M. anisopliae* treatment or control group.

Samples	Total_Reads	Mapped_Reads	Mapped_reads(+)	Mapped_reads(-)
T16-1	19529484	12745435(65.26%)	11487368(58.82%)	1258067(6.44%)
T16-2	23076357	15196366(65.85%)	13674474(59.26%)	1521892(6.60%)
T16-3	22215651	14522239(65.37%)	12866299(57.92%)	1655940(7.45%)
T24-1	20830285	13067200(62.73%)	11602217(55.70%)	1464983(7.03%)
T24-2	18653498	11814884(63.34%)	10268381(55.05%)	1546503(8.29%)
T24-3	19393380	12847411(66.25%)	11446016(59.02%)	1401395(7.23%)
T4-1	24624901	15610327(63.39%)	13652182(55.44%)	1958145(7.95%)
T4-2	22581238	14303883(63.34%)	13077462(57.91%)	1226421(5.43%)
T4-3	26008902	16682869(64.14%)	15283744(58.76%)	1399125(5.38%)
T8-1	13063791	7718660(59.08%)	6481391(49.61%)	1237269(9.47%)
T8-2	16190392	10375403(64.08%)	9570174(59.11%)	805229(4.97%)
T8-3	20114536	13438683(66.81%)	12183210(60.57%)	1255473(6.24%)
W16-1	17050032	10964021(64.30%)	9443457(55.39%)	1520564(8.92%)
W16-2	15561907	10615678(68.22%)	9839645(63.23%)	776033(4.99%)
W16-3	14742125	9320613(63.22%)	8097705(54.93%)	1222908(8.30%)
W24-1	29854390	19445468(65.13%)	16851712(56.45%)	2593756(8.69%)
W24-2	22888958	14531063(63.49%)	12497847(54.60%)	2033216(8.88%)
W24-3	25603302	15729349(61.43%)	13313447(52.00%)	2415902(9.44%)
W4-1	15110103	9716832(64.31%)	9109922(60.29%)	606910(4.02%)
W4-2	13117631	7933804(60.48%)	7416330(56.54%)	517474(3.94%)
W4-3	13208019	7816533(59.18%)	7287902(55.18%)	528631(4.00%)
W8-1	19715790	12432256(63.06%)	11173276(56.67%)	1258980(6.39%)
W8-2	15690068	9815680(62.56%)	8960483(57.11%)	855197(5.45%)
W8-3	16484890	10372039(62.92%)	9145117(55.48%)	1226922(7.44%)

T, the fungal *M. anisopliae*-infected treatment; W, Control group.

Table S4. Top ten up- and down-regulated DEmiRNAs in *N. lugens* after *M. anisopliae* challenge.

Treatment	Gene ID	P-value	Fold Change (log2)	Up/Down regulated
4 h after infection	NL-miR-1676	1.42E-05	1.67	Up
	NL-miR-2333	0.011078	1.41	Up
	NL-miR-1377	0.006959	1.40	Up
	NL-miR-631	0.002445	1.26	Up
	NL-miR-1047	0.000886	1.22	Up
	NL-miR-156	0.017649	1.22	Up
	NL-miR-2380	0.021169	1.21	Up
	NL-miR-270	0.008357	1.21	Up
	NL-miR-1439	0.008357	1.21	Up
	NL-miR-1864	0.001419	1.15	Up
	NL-miR-918	0.001259	-1.62	down
	NL-miR-184	0.004441	-1.48	down
	NL-miR-565	0.016421	-1.32	down
	NL-miR-597	0.020062	-1.29	down
	NL-miR-1511	0.010315	-1.19	down
	NL-miR-2191	0.019404	-1.16	down
	NL-miR-609	0.001103	-1.14	down
	NL-miR-1124	0.029833	-1.13	down
	NL-miR-1764	0.001694	-1.13	down
	NL-miR-957	0.033025	-1.09	down
8 h after infection	NL-miR-940	0.000571	2.20	up
	NL-miR-2297	0.000272	2.20	up
	NL-miR-1343	0.000208	2.15	up
	NL-miR-1651	0.001157	2.10	up
	NL-miR-260	5.02E-08	2.04	up

	NL-miR-1675	0.00487	1.96	up
	NL-miR-2180	0.01550	1.58	up
	NL-miR-1972	0.03142	1.50	up
	NL-miR-2235	0.02841	1.49	up
	NL-miR-2067	0.01386	1.46	up
	NL-miR-1380	0.00019	-2.32	down
	NL-miR-682	2.60E-05	-2.24	down
	NL-miR-1865	0.00077	-1.82	down
	NL-miR-2028	0.00962	-1.63	down
	NL-miR-58	0.00962	-1.63	down
	NL-miR-1252	0.00657	-1.63	down
	NL-miR-490	0.01905	-1.61	down
	NL-miR-1404	7.26E-05	-1.61	down
	NL-miR-1238	0.02242	-1.59	down
	NL-miR-1071	0.00971	-1.53	down
16 h after infection	NL-miR-1982	0.00028	2.42	up
	NL-miR-1147	5.47E-08	2.12	up
	NL-miR-453	0.00491	2.08	up
	NL-miR-1462	1.28E-07	2.08	up
	NL-miR-1124	2.06E-07	2.04	up
	NL-miR-754	0.00058	1.97	up
	NL-miR-1056	2.38E-06	1.96	up
	NL-miR-982	2.38E-06	1.96	up
	NL-miR-715	0.00106	1.94	up
	NL-miR-2191	3.45E-07	1.94	up
	NL-miR-274	0.00078	-2.44	down
	NL-miR-1040	0.00031	-2.17	down
	NL-miR-983	0.00031	-2.17	down
	NL-miR-29	6.05E-05	-1.84	down
	NL-miR-2233	0.00375	-1.82	down
	NL-miR-1834	4.57E-05	-1.71	down
	NL-miR-1678	0.02696	-1.65	down

	NL-miR-1673	0.01980	-1.61	down
	NL-miR-1714	0.01980	-1.61	down
	NL-miR-683	0.00742	-1.61	down
24 h after infection	NL-miR-54	3.58E-05	2.05	up
	NL-miR-940	2.89E-05	1.98	up
	NL-miR-490	7.98E-07	1.64	up
	NL-miR-1546	0.00016	1.40	up
	NL-miR-2298	0.00016	1.40	up
	NL-miR-457	0.00016	1.40	up
	NL-miR-612	0.00016	1.40	up
	NL-miR-2038	0.00510	1.35	up
	NL-miR-756	0.00836	1.33	up
	NL-miR-1058	0.00983	1.30	up
	NL-miR-1927	5.08E-05	-1.86	down
	NL-miR-2301	0.00022	-1.39	down
	NL-miR-359	0.00022	-1.39	down
	NL-miR-430	0.00551	-1.39	down
	NL-miR-1136	0.00267	-1.33	down
	NL-miR-1257	0.00127	-1.24	down
	NL-miR-153	0.00438	-1.18	down
	NL-miR-931	0.00849	-1.16	down
	NL-miR-1094	0.01998	-1.14	down
	NL-miR-1377	0.00495	-1.14	down

Table S5. The number information of annotated targets of miRNAs with different database.

Database use	Number of target mRNAs	300 ≤ length < 1000 (number)	length ≥ 1000 (number)
COG database	3453	393	3055
GO database	4360	688	3652
KEGG database	5468	733	4723
KOG database	7581	1016	6547
Pfam database	8782	1211	7555
Swissprot database	6800	870	5914
eggNOG database	9827	1502	8302
Nr Annotation	11044	1967	9048
All Annotated targets	11046	1969	9048

Table S6. The identification of pathways, target genes of *N. lugens* miRNAs after fungal infection during different times.

Post infection	Pathways	KO_ID	Number of targets	Identification of target genes	KEEGs
4 hours	Peroxisome	ko04146	6	gene1407;gene7007;gene18035;gene15312;gene21074;gene6018	K00106+K00106+K00624+K13356+K13356+K12261
	Carbon metabolism	ko01200	10	gene5332;gene7933;gene20873;gene15227;gene11896;gene16451;gene3441;gene15818;gene2043;gene14573	K00164+K00164+K00033+K01895+K00234+K00140+K00281+K00261+K00261+K00605
	RNA degradation	ko03018	7	gene5953;gene16105;gene4570;gene4831;gene2107;gene1028;gene17448	K12604+K12603+K12607+K10643+K00850+K12600+K00850
	Glycerophospholipid metabolism	ko00564	2	gene362;gene791	K14156+K08729
	Basal transcription factors	ko03022	3	gene16090;gene8238;gene6731	K03125+K03125+K03131
	Lysine degradation	ko00310	10	gene6127;gene6663;gene20335;gene8720;gene13961;gene8228;gene1750;gene10527;gene6327;gene4263	K09186+K09188+K11419+K09189+K11424+K11424+K11424+K11424+K11424+K11422
	Phototransduction - fly	ko04745	6	gene13232;gene6073;gene10149;gene13822;gene5881;gene4081	K13805+K13805+K13803+K08834+K00910+K04634
	Oxidative phosphorylation	ko00190	3	gene6358;gene11049;gene15673	K00411+K03953+K11352
	TGF-beta signaling pathway	ko04350	6	gene10115;gene11452;gene14820;gene11453;gene12537;gene19182	K04679+K04498+K04679+K04498+K04498+K03456

RNA transport	ko03013	18	gene15455;gene6640;gene11186;gene18626;gene4593;gene3178;gene18927;gene16742;gene15934;gene14023;gene9672;gene19738;gene6572;gene3430;gene11878;gene9978;gene14005;gene9512	K09291+K12172+K14296+K03254+K05749+K14309+K00784+K14307+K03260+K14297+K14312+K03254+K03251+K02516+K03248+K03246+K14319+K14313
ECM-receptor interaction	ko04512	4	gene13844;gene17671;gene3629;gene18353	K06254+K05637+K05719+K05637
Endocytosis	ko04144	7	gene892;gene12596;gene2747;gene11912;gene11730;gene2208;gene971	K18443+K04707+K12491+K12486+K10591+K12475+K12470
Fatty acid metabolism	ko01212	3	gene5274;gene4724;gene15706	K00665+K00507+K00249
MAPK signaling pathway - fly	ko04013	7	gene14455;gene9815;gene18032;gene11296;gene12904;gene5001;gene18069	K05088+K20225+K08545+K08545+K08601+K07293+K08601
Ubiquitin mediated proteolysis	ko04120	18	gene441;gene4577;gene10524;gene13968;gene14430;gene18292;gene7191;gene477;gene1894;gene18390;gene939;gene13209;gene10007;gene5649;gene8711;gene2595;gene11987;gene16412	K10581+K10589+K10592+K03348+K10594+K03364+K03364+K10592+K10593+K10593+K10605+K10685+K10595+K03350+K10592+K10588+K10260+K10592
Wnt signaling pathway	ko04310	7	gene8624;gene8811;gene11056;gene69;gene5149;gene6039;gene1945	K03068+K00444+K17388+K04511+K02432+K04491+K04348
Phagosome	ko04145	2	gene19325;gene16867	K02154+K00914
Glycosylphosphatidylinositol(GPI)-anchor	ko00563	3	gene1026;gene19755;gene20373	K05288+K08098+K08098

biosynthesis				
Steroid biosynthesis	ko00100	4	gene7967;gene15740;gene11481;gene6108	K00213+K00213+K00213+K07750
FoxO signaling pathway	ko04068	7	gene13747;gene16512;gene14986;gene1805;gene7151;gene16251;gene7182	K05868+K01596+K04402+K06631+K11838+K11838+K09385
Non-homologous end-joining	ko03450	2	gene18894;gene18953	K10866+K10885
RNA polymerase	ko03020	5	gene19306;gene4308;gene6502;gene6896;gene202	K03004+K03010+K03023+K03006+K03014
Insect hormone biosynthesis	ko00981	2	gene10544;gene14413	K14985+K14939
Circadian rhythm - fly	ko04711	3	gene17662;gene6557;gene5338	K12074+K12074+K02223
ABC transporters	ko02010	3	gene2956;gene7573;gene10513	K05648+K05665+K05673
Hippo signaling pathway - fly	ko04391	9	gene2114;gene19839;gene10450;gene2144;gene14207;gene2634;gene13422;gene2080;gene12126	K16175+K16681+K16681+K16677+K03456+K16680+K16676+K05628+K04237
Fanconi anemia pathway	ko03460	3	gene14719;gene7398;gene3826	K10896+K02350+K10858
Ribosome	ko03010	5	gene10516;gene17400;gene4162;gene20881;gene13692	K02917+K02988+K02981+K02993+K02993
Starch and sucrose metabolism	ko00500	2	gene1370;gene15883	K00700+K16055
Lysosome	ko04142	7	gene8809;gene10477;gene4207;gene6229;gene9001;gene3785;gene5931	K12382+K12350+K01363+K12391+K12311+K01192+K01204

Neuroactive ligand-receptor interaction	ko04080	6	gene12120;gene19681;gene15564;gene8564;gene7423;gene887	K04153+K04153+K05313+K14049+K04239+K04209
Nucleotide excision repair	ko03420	3	gene3678;gene7471;gene7100	K02324+K10846+K02202
mTOR signaling pathway	ko04150	7	gene4295;gene2953;gene9821;gene395;gene10398;gene6992;gene4180	K07204+K06276+K20410+K03258+K20397+K03259+K08269
Ribosome biogenesis in eukaryotes	ko03008	9	gene3213;gene1201;gene3188;gene13566;gene10421;gene18852;gene1164;gene13568;gene19558	K14570+K14572+K14569+K07179+K14572+K14567+K14554+K07179+K14536
Proteasome	ko03050	2	gene4368;gene18402	K03065+K03036
Purine metabolism	ko00230	2	gene12584;gene14837	K11265+K12319
mRNA surveillance pathway	ko03015	6	gene16716;gene11041;gene1371;gene312;gene973;gene13214	K14409+K03267+K14408+K11584+K13171+K14411
Adrenergic signaling in cardiomyocytes	ko04261	1	gene19268	K11584
DNA replication	ko03030	7	gene7027;gene2987;gene16807;gene548;gene4224;gene15139;gene11015	K02212+K02685+K02541+K02542+K10742+K02320+K02541
Glutathione metabolism	ko00480	4	gene2053;gene19263;gene14752;gene14755	K11205+K01581+K01255+K01255
Biosynthesis of amino acids	ko01230	10	gene10466;gene18989;gene2372;gene3186;gene18220;gene9799;gene16651;gene17539;gene7644;gene15722	K12657+K01694+K01681+K00264+K00826+K00826+K00600+K01803+K17989+K14677
Spliceosome	ko03040	12	gene2773;gene1042;gene2462;gene5557;	K12823+K12828+K12815+K12741+K1282

				gene10409;gene14275;gene5536;gene7516;gene2823;gene1991;gene9570;gene11142	6+K12867+K12896+K12891+K12897+K12741+K12841+K12837
	Protein processing in endoplasmic reticulum	ko04141	14	gene12477;gene3539;gene2198;gene2460;gene9177;gene3049;gene5395;gene9659;gene7748;gene9308;gene2692;gene4696;gene7718;gene6790	K09486+K14008+K16196+K09530+K10601+K14011+K10086+K09054+K10950+K09502+K09517+K09487+K12275+K09487
	Notch signaling pathway	ko04330	6	gene18280;gene10215;gene368;gene13974;gene19276;gene8074	K06052+K06052+K02599+K06067+K06053+K04505
8 hours	Lysosome	ko04142	8	gene8809;gene10477;gene12057;gene19020;gene15183;gene18854;gene5575;gene9197	K12382+K12350+K12396+K12392+K12373+K01279+K01136+K20730
	Drug metabolism - other enzymes	ko00983	2	gene12730;gene13089	K01951+K00699
	Fatty acid metabolism	ko01212	6	gene5036;gene11115;gene466;gene19069;gene19167;gene5811	K00665+K00645+K00507+K10703+K10256+K15013
	Dorso-ventral axis formation	ko04320	5	gene16145;gene7205;gene3499;gene10493;gene20272	K02184+K02156+K02359+K02602+K03211
	Neuroactive ligand-receptor interaction	ko04080	10	gene12631;gene2149;gene3574;gene14228;gene5467;gene2089;gene14319;gene4522;gene4;gene16416	K05313+K04280+K01312+K05213+K04131+K14049+K04209+K05273+K05175+K04157
	mTOR signaling pathway	ko04150	9	gene15239;gene12863;gene4295;gene13483;gene7932;gene395;gene13299;gene1	K08267+K03875+K07204+K13780+K02150+K03258+K07207+K14299+K08266

			6173;gene9240	
FoxO signaling pathway	ko04068	1	gene13747	K05868
Ubiquitin mediated proteolysis	ko04120	23	gene441;gene4577;gene10524;gene7160;gene7792;gene7191;gene477;gene4127;gene18497;gene2137;gene18390;gene5649;gene18639;gene17670;gene11200;gene4520;gene11302;gene8711;gene17949;gene2022;gene2595;gene16638;gene12505	K10581+K10589+K10592+K03348+K03178+K03364+K10592+K10608+K10598+K03348+K10593+K03350+K16061+K10587+K04706+K10595+K10610+K10592+K10593+K10593+K10588+K10594+K10585
MAPK signaling pathway - fly	ko04013	9	gene14455;gene4299;gene10172;gene9815;gene16584;gene13064;gene6246;gene12174;gene778	K05088+K12379+K20210+K20225+K13411+K08840+K14290+K13411+K12379
Folate biosynthesis	ko00790	4	gene435;gene12436;gene3017;gene1580	K01077+K01077+K01077+K01077
Amino sugar and nucleotide sugar metabolism	ko00520	6	gene2155;gene8513;gene19784;gene5145;gene16693;gene13628	K01183+K00326+K00326+K00698+K00326+K01183
RNA polymerase	ko03020	5	gene19306;gene8041;gene6896;gene4198;gene6894	K03004+K03013+K03006+K03018+K03006
Aminoacyl-tRNA biosynthesis	ko00970	2	gene17918;gene1060	K01869+K01873
Spliceosome	ko03040	15	gene17077;gene10243;gene2773;gene12993;gene269;gene19174;gene10409;gene3905;gene18354;gene17045;gene6027;gene5529;gene14100;g	K11092+K11092+K12823+K12856+K12854+K12874+K12826+K12838+K12874+K12872+K12811+K12831+K12823+K12878+K12855

			ene19175;gene12822	
RNA degradation	ko03018	11	gene5953;gene7389;gene1022;gene16013;gene18863;gene18824;gene4822;gene9557;gene2107;gene13118;gene5292	K12604+K12619+K12598+K04043+K11600+K12590+K12580+K03678+K00850+K12606+K12599
Terpenoid backbone biosynthesis	ko00900	3	gene8436;gene15977;gene5289	K00021+K00787+K08658
Phototransduction - fly	ko04745	5	gene4712;gene13232;gene6073;gene11684;gene575	K13806+K13805+K13805+K08834+K04547
Phagosome	ko04145	8	gene6249;gene3532;gene1297;gene14443;gene3118;gene17575;gene7203;gene2438	K10413+K00921+K07374+K07374+K07374+K07375+K07375+K07375
Lysine degradation	ko00310	14	gene6663;gene3750;gene8720;gene13961;gene8228;gene1750;gene10527;gene6327;gene11119;gene4263;gene18804;gene19468;gene11538;gene16272	K09188+K11423+K09189+K11424+K11424+K11424+K11424+K11424+K14157+K11422+K11424+K11424+K11424+K11424
TGF-beta signaling pathway	ko04350	5	gene11452;gene3360;gene10082;gene229;gene12537	K04498+K04681+K04662+K04678+K04498
Phosphatidylinositol signaling system	ko04070	9	gene18679;gene15865;gene1762;gene4320;gene17457;gene10872;gene1569;gene2670;gene15606	K00923+K00901+K00901+K00888+K00901+K00901+K00911+K01107+K01092
Glycerophospholipid metabolism	ko00564	5	gene18178;gene15669;gene13633;gene18058;gene20234	K00111+K14676+K01049+K01049+K01049
Basal transcription factors	ko03022	4	gene8238;gene8384;gene12773;gene4122	K03125+K03124+K14650+K03128
Glycosami	ko00534	4	gene19203;gene664	K07809+K02514+K0

noglycan biosynthesis - heparan sulfate / heparin			2;gene14129;gene17634	2366+K02577
Protein processing in endoplasmic reticulum	ko04141	13	gene12477;gene2018;gene2198;gene8718;gene1040;gene2106;gene7866;gene7748;gene871;gene7282;gene15671;gene6790;gene10435	K09486+K14006+K16196+K11718+K08852+K14010+K09485+K10950+K09523+K10597+K13249+K09487+K09521
Endocytosis	ko04144	7	gene6694;gene6841;gene13361;gene4409;gene3942;gene13955;gene291	K12494+K18442+K11824+K04646+K12479+K12488+K19367
RNA transport	ko03013	12	gene15859;gene4999;gene6640;gene15046;gene20349;gene2464;gene956;gene17151;gene17679;gene11267;gene16301;gene1066	K00784+K14310+K12172+K14317+K14288+K09291+K14293+K03238+K03253+K14310+K03680+K02516
Notch signaling pathway	ko04330	4	gene10215;gene368;gene14547;gene8075	K06052+K02599+K04496+K04496
Fanconi anemia pathway	ko03460	8	gene2679;gene7398;gene19586;gene17701;gene432;gene7321;gene2579;gene13987	K10895+K02350+K10891+K10891+K03165+K10901+K08775+K08775
Peroxisome	ko04146	8	gene21312;gene3843;gene10702;gene1190;gene11116;gene12642;gene19361;gene15886	K03781+K01640+K13356+K13343+K13356+K13342+K13345+K00106
DNA replication	ko03030	2	gene7027;gene3895	K02212+K03469
ECM-receptor interaction	ko04512	3	gene1614;gene15875;gene18535	K19719+K05635+K06240
Purine metabolism	ko00230	4	gene6538;gene16710;gene3321;gene938	K12323+K01769+K13293+K13298

			2	
Glutathione metabolism	ko00480	3	gene9482;gene3097;gene5089	K11140+K11142+K11140
Arginine and proline metabolism	ko00330	2	gene20083;gene7919	K00819+K00294
Ribosome	ko03010	4	gene10516;gene15123;gene19521;gene2324	K02917+K02925+K02894+K02893
Hippo signaling pathway - fly	ko04391	9	gene2114;gene10450;gene2144;gene2634;gene11876;gene2080;gene10736;gene12467;gene12941	K16175+K16681+K16677+K16680+K05628+K05628+K12076+K03209+K02306
ABC transporters	ko02010	4	gene10513;gene7632;gene14331;gene3494	K05673+K05662+K05641+K05658
Carbon metabolism	ko01200	5	gene4852;gene7934;gene2814;gene15818;gene2043	K01965+K00164+K00844+K00261+K00261
Ribosome biogenesis in eukaryotes	ko03008	11	gene9827;gene19487;gene15155;gene17484;gene16305;gene6733;gene12547;gene11277;gene19874;gene13540;gene19181	K14557+K14575+K14571+K14575+K06943+K14550+K14575+K14571+K14521+K06943+K03264
Biosynthesis of amino acids	ko01230	10	gene2316;gene18989;gene2372;gene3186;gene8428;gene4970;gene18331;gene21139;gene1048;gene1046	K00030+K01694+K01681+K00264+K00873+K00058+K01702+K00927+K00264+K00264
Glycosylphosphatidylinositol(GPI)-anchor biosynthesis	ko00563	2	gene1340;gene1026	K05294+K05288
mRNA surveillance pathway	ko03015	13	gene15204;gene12035;gene7414;gene2600;gene11041;gene8572;gene19324;gene	K08873+K15542+K14400+K11124+K03267+K00565+K14403+K14962+K14398+K1

			18553;gene14356;gene650;gene19623;gene7179;gene15915	3114+K14404+K08873+K14405
	Proteasome	ko03050	4	gene11024;gene7373;gene8098;gene506	K03032+K02737+K03037+K06699
	Starch and sucrose metabolism	ko00500	5	gene1370;gene1385;gene19998;gene15883;gene10969	K00700+K01196+K05349+K16055+K00693
	Wnt signaling pathway	ko04310	6	gene7867;gene8624;gene3662;gene10194;gene12466;gene10334	K03068+K03068+K10151+K04510+K01064+K02842
16 hours	Biosynthesis of amino acids	ko01230	10	gene9242;gene10466;gene2372;gene3186;gene4970;gene15874;gene7644;gene15722;gene14916;gene11695	K12657+K12657+K01681+K00264+K00058+K01915+K17989+K14677+K00058+K14454
	Spliceosome	ko03040	22	gene2773;gene1042;gene6435;gene269;gene14275;gene5536;gene7516;gene3229;gene9485;gene9487;gene17045;gene15453;gene3071;gene360;gene7409;gene18607;gene14839;gene12822;gene833;gene7646;gene11142;gene17201	K12823+K12828+K12833+K12854+K12867+K12896+K12891+K12818+K12825+K12825+K12872+K12815+K12823+K12880+K12826+K12816+K12840+K12855+K12821+K12880+K12837+K12890
	Basal transcription factors	ko03022	4	gene16090;gene8238;gene9161;gene18949	K03125+K03125+K03138+K14535
	Aminoacyl-tRNA biosynthesis	ko00970	3	gene10866;gene13801;gene17441	K14163+K01869+K14163
	Folate biosynthesis	ko00790	4	gene435;gene12436;gene11652;gene21142	K01077+K01077+K01307+K01307
	Amino sugar and nucleotide	ko00520	5	gene2155;gene13900;gene7456;gene13628;gene13457	K01183+K01183+K01183+K01183+K01639

sugar metabolism				
Tryptophan metabolism	ko00380	4	gene2303;gene20092;gene16748;gene5790	K00502+K00502+K00252+K01593
Peroxisome	ko04146	10	gene17260;gene1407;gene7007;gene18035;gene1190;gene13231;gene6563;gene19726;gene15115;gene6018	K00106+K00106+K00106+K00624+K13343+K13341+K05940+K13356+K12405+K12261
Purine metabolism	ko00230	5	gene16710;gene3321;gene15302;gene14837;gene15029	K01769+K13293+K12323+K12319+K00939
TGF-beta signaling pathway	ko04350	8	gene10115;gene11452;gene14820;gene229;gene11453;gene12537;gene8402;gene15205	K04679+K04498+K04679+K04678+K04498+K04498+K04657+K04498
Hippo signaling pathway - fly	ko04391	18	gene4601;gene2114;gene19839;gene10450;gene7298;gene2144;gene2634;gene11876;gene831;gene2080;gene13702;gene12467;gene14995;gene11892;gene14775;gene14773;gene12126;gene3280	K06095+K16175+K16681+K16681+K04676+K16677+K16680+K05628+K06094+K05628+K16674+K03209+K03209+K16677+K16175+K06626+K04237+K16673
RNA polymerase	ko03020	6	gene19306;gene4308;gene6896;gene4198;gene16982;gene3336	K03004+K03010+K03006+K03018+K03027+K03027
Carbon metabolism	ko01200	14	gene5332;gene7933;gene15227;gene11896;gene16451;gene2814;gene19172;gene20125;gene15818;gene2043;gene14573;gene19109;gene8062;gene15390	K00164+K00164+K01895+K00234+K00140+K00844+K01900+K19269+K00261+K00261+K00605+K00844+K01053+K01810
Ribosome biogenesis	ko03008	16	gene2243;gene3213;gene1201;gene3188;	K14572+K14570+K14572+K14569+K1457

in eukaryotes			gene10421;gene15155;gene6733;gene12547;gene11277;gene1164;gene18942;gene21140;gene15324;gene19181;gene19558;gene6732	2+K14571+K14550+K14575+K14571+K14554+K14544+K14569+K14536+K03264+K14536+K14550
RNA degradation	ko03018	17	gene6695;gene5953;gene7389;gene16105;gene4570;gene4831;gene11265;gene2107;gene13118;gene565;gene1028;gene17448;gene11828;gene5890;gene1616;gene8660;gene7971	K12571+K12604+K12619+K12603+K12607+K10643+K12572+K00850+K12606+K01689+K12600+K00850+K13126+K12618+K01148+K12606+K12572
Glycosylphosphatidylinositol(GPI)-anchor biosynthesis	ko00563	6	gene1340;gene12400;gene20451;gene19755;gene20373;gene8814	K05294+K09658+K08098+K08098+K08098+K05287
FoxO signaling pathway	ko04068	8	gene13747;gene16512;gene14986;gene1805;gene7151;gene16251;gene7182;gene12663	K05868+K01596+K04402+K06631+K11838+K11838+K09385+K10305
Neuroactive ligand-receptor interaction	ko04080	17	gene2121;gene10780;gene8766;gene12120;gene6614;gene19681;gene18133;gene5479;gene4;gene2264;gene7423;gene14131;gene8761;gene12028;gene16632;gene7;gene887	K04134+K05208+K05210+K04153+K05210+K04153+K04209+K04209+K05175+K04577+K04239+K04280+K04209+K04209+K04209+K05181+K04209
Phototransduction - fly	ko04745	10	gene13232;gene10150;gene5888;gene18811;gene6073;gene1615;gene11684;gene15882;gene5881;gene19393	K13805+K04967+K08834+K08834+K13805+K05692+K08834+K05692+K00910+K04967
Proteasome	ko03050	7	gene4368;gene1840	K03065+K03036+K0

			2;gene277;gene11024;gene19505;gene2793;gene10446	3062+K03032+K03030+K03030+K03066
Phagosome	ko04145	9	gene19325;gene1297;gene6926;gene17575;gene7203;gene11286;gene9608;gene16867;gene17147	K02154+K07374+K07375+K07375+K07375+K07374+K12182+K00914+K02146
DNA replication	ko03030	5	gene7027;gene16807;gene4224;gene15139;gene11015	K02212+K02541+K10742+K02320+K02541
Wnt signaling pathway	ko04310	9	gene11056;gene69;gene5149;gene6039;gene14778;gene10194;gene3571;gene1847;gene16229	K17388+K04511+K02432+K04491+K02157+K04510+K04468+K02105+K02222
Nucleotide excision repair	ko03420	5	gene3678;gene7471;gene9837;gene19984;gene7100	K02324+K10846+K10841+K10846+K02202
Protein processing in endoplasmic reticulum	ko04141	20	gene12477;gene12059;gene7102;gene14191;gene2198;gene2460;gene4443;gene3049;gene20905;gene1040;gene5395;gene9234;gene7282;gene6961;gene17209;gene4696;gene7718;gene6790;gene6972;gene13047	K09486+K14005+K04523+K14007+K16196+K09530+K07151+K14011+K14020+K08852+K10086+K08852+K10597+K04554+K04079+K09487+K12275+K09487+K09584+K14026
Lysosome	ko04142	12	gene8809;gene10477;gene3853;gene9002;gene8195;gene4207;gene6229;gene5796;gene9197;gene3871;gene7175;gene2248	K12382+K12350+K12397+K12311+K12397+K01363+K12391+K12347+K20730+K12373+K01158+K12307
Fatty acid elongation	ko00062	2	gene17780;gene12262	K10249+K10249
Phosphatidylinositol signaling system	ko04070	12	gene13503;gene18679;gene16205;gene8523;gene2017;gene10872;gene16252;gen	K13024+K00923+K18083+K00888+K13711+K00901+K20278+K20279+K01107+K2

			e18915;gene2670;gene16718;gene17453;gene3842	0279+K20279+K00920
Fanconi anemia pathway	ko03460	7	gene2679;gene14719;gene7398;gene432;gene2579;gene13987;gene16433	K10895+K10896+K02350+K03165+K08775+K08775+K13960
Ubiquitin mediated proteolysis	ko04120	32	gene441;gene7317;gene4577;gene10524;gene13968;gene7160;gene14430;gene18292;gene7191;gene477;gene4127;gene18497;gene1894;gene18390;gene939;gene4172;gene13209;gene10007;gene5584;gene17670;gene14227;gene11200;gene17949;gene2022;gene13508;gene2595;gene16638;gene11987;gene15991;gene5854;gene16412;gene11551	K10581+K10599+K10589+K10592+K03348+K03348+K10594+K03364+K03364+K10592+K10608+K10598+K10593+K10593+K10605+K10590+K10685+K10595+K10587+K10587+K10587+K04706+K10593+K10593+K10593+K10588+K10594+K10260+K10576+K10686+K10592+K10570
Ribosome	ko03010	6	gene17400;gene4162;gene7405;gene14294;gene7158;gene13970	K02988+K02981+K02936+K02976+K02966+K02966
MAPK signaling pathway - fly	ko04013	15	gene14455;gene9815;gene16584;gene12904;gene8764;gene11156;gene11867;gene13064;gene13315;gene16661;gene12174;gene18069;gene16583;gene11822;gene9490	K05088+K20225+K13411+K08601+K17536+K04428+K14290+K08840+K08840+K05088+K13411+K08601+K13411+K17512+K04461
Endocytosis	ko04144	18	gene892;gene12596;gene1332;gene2747;gene19144;gene11912;gene11730;gene2208;gene3945;gene4409;gene9052;gene1	K18443+K04707+K18462+K12491+K18468+K12486+K10591+K12475+K10396+K04646+K18464+K05754+K18442+K12488+

			6234;gene18715;gene13955;gene10994;gene14050;gene18829;gene20428	K11839+K05747+K11825+K05093
mTOR signaling pathway	ko04150	10	gene15239;gene4295;gene2953;gene9821;gene11595;gene395;gene13299;gene6051;gene16292;gene14860	K08267+K07204+K06276+K20410+K02145+K03258+K07207+K01110+K02147+K02147
RNA transport	ko03013	31	gene15859;gene4346;gene6640;gene10928;gene20050;gene18626;gene9120;gene15046;gene3178;gene6518;gene18927;gene16742;gene15934;gene20349;gene9672;gene18180;gene8457;gene19042;gene12322;gene956;gene17151;gene17629;gene19738;gene3430;gene9978;gene14005;gene8077;gene16301;gene9512;gene3258;gene7864	K00784+K14314+K12172+K03260+K03254+K03254+K03243+K14317+K14309+K13137+K00784+K14307+K03260+K14288+K14312+K13133+K03231+K03231+K03231+K14293+K03238+K14305+K03254+K02516+K03246+K14319+K03262+K03680+K14313+K02516+K14295
Glutathione metabolism	ko00480	5	gene2053;gene6615;gene14752;gene14755;gene3197	K11205+K11140+K01255+K01255+K00681
Lysine degradation	ko00310	15	gene6127;gene6663;gene21305;gene3750;gene8720;gene13961;gene8228;gene1750;gene10527;gene6327;gene2748;gene18804;gene21161;gene19773;gene19468	K09186+K09188+K11433+K11423+K09189+K11424+K11424+K11424+K11424+K11424+K11422+K11424+K11424+K06101+K11424
Oxidative phosphorylation	ko00190	5	gene6358;gene3762;gene14900;gene15673;gene9028	K00411+K03934+K03951+K11352+K02128
mRNA surveillanc	ko03015	17	gene7414;gene16716;gene11041;gene13	K14400+K14409+K03267+K14408+K1158

	e pathway			71;gene312;gene6536;gene19324;gene16532;gene650;gene13701;gene1316;gene4825;gene3174;gene7179;gene652;gene8096;gene735	4+K11584+K14403+K14416+K13114+K11583+K14406+K08873+K14376+K08873+K13114+K14284+K14397
	Notch signaling pathway	ko04330	5	gene18280;gene10215;gene368;gene13974;gene6550	K06052+K06052+K02599+K06067+K06053
	ABC transporters	ko02010	7	gene13649;gene10513;gene3804;gene13535;gene14331;gene9783;gene3494	K05674+K05673+K05641+K05665+K05641+K05662+K05658
	Starch and sucrose metabolism	ko00500	4	gene1370;gene14278;gene1385;gene2201	K00700+K01194+K01196+K16055
	ECM-receptor interaction	ko04512	6	gene13844;gene17671;gene3629;gene18353;gene7134;gene13778	K06254+K05637+K05719+K05637+K05719+K06240
	Arginine and proline metabolism	ko00330	6	gene20083;gene2047;gene11005;gene15531;gene9528;gene15135	K00819+K01611+K00472+K00472+K00933+K00819
24 hours	RNA polymerase	ko03020	3	gene4308;gene6896;gene4198	K03010+K03006+K03018
	Dorso-ventral axis formation	ko04320	6	gene14815;gene1868;gene11350;gene7566;gene7130;gene4084	K03099+K02602+K02602+K04361+K02156+K04364
	Ubiquitin mediated proteolysis	ko04120	17	gene441;gene7317;gene4577;gene10524;gene13968;gene7160;gene14430;gene477;gene4172;gene10007;gene5527;gene11200;gene18703;gene2257;gene2595;gene4563;gene11551	K10581+K10599+K10589+K10592+K03348+K03348+K10594+K10592+K10590+K10595+K10596+K04706+K10583+K04552+K10588+K07868+K10570
	Purine metabolism	ko00230	4	gene16710;gene3321;gene14837;gene9316	K01769+K13293+K12319+K13755

Ribosome biogenesis in eukaryotes	ko03008	11	gene3188;gene10421;gene15155;gene16305;gene12547;gene11277;gene13540;gene9458;gene1164;gene19181;gene4846	K14569+K14572+K14571+K06943+K14575+K14571+K06943+K14556+K14554+K03264+K14552
Lysosome	ko04142	12	gene8809;gene10477;gene3853;gene9002;gene8195;gene12057;gene9893;gene10113;gene8646;gene18854;gene1117;gene17153	K12382+K12350+K12397+K12311+K12397+K12396+K12404+K01444+K01374+K01279+K01379+K01205
Amino sugar and nucleotide sugar metabolism	ko00520	5	gene2155;gene8513;gene19784;gene13628;gene15868	K01183+K00326+K00326+K01183+K01784
Glycosaminoglycan biosynthesis - heparan sulfate / heparin	ko00534	3	gene19203;gene13645;gene14129	K07809+K00771+K02366
mRNA surveillance pathway	ko03015	18	gene7414;gene2600;gene12936;gene16716;gene15893;gene16532;gene6048;gene13701;gene4825;gene3174;gene315;gene13293;gene442;gene19037;gene14668;gene2384;gene5781;gene735	K14400+K11124+K14326+K14409+K14327+K14416+K06100+K11583+K08873+K14376+K03265+K06269+K06269+K06269+K14323+K14403+K14401+K14397
TGF-beta signaling pathway	ko04350	9	gene10115;gene11452;gene14820;gene10082;gene229;gene11453;gene12537;gene13159;gene15205	K04679+K04498+K04679+K04662+K04678+K04498+K04498+K13578+K04498
Biosynthesis of amino acids	ko01230	12	gene2316;gene9242;gene13663;gene18228;gene3186;gene8902;gene7762;gene49	K00030+K12657+K12657+K01623+K00264+K01623+K00789+K00058+K01958+K1

			70;gene17068;gene7644;gene13228;gene14916	7989+K01758+K00058
RNA degradation	ko03018	12	gene6695;gene5953;gene4570;gene4831;gene8421;gene1022;gene2107;gene13118;gene5635;gene1028;gene17448;gene11828	K12571+K12604+K12607+K10643+K12585+K12598+K00850+K12606+K12614+K12600+K00850+K13126
Alanine, aspartate and glutamate metabolism	ko00250	2	gene7287;gene14539	K11540+K11540
Spliceosome	ko03040	19	gene12993;gene2462;gene10864;gene6435;gene269;gene19174;gene3905;gene15453;gene19300;gene7409;gene7183;gene6656;gene8903;gene4761;gene17150;gene19661;gene12822;gene7646;gene4026	K12856+K12815+K12874+K12833+K12854+K12874+K12838+K12815+K12818+K12826+K12830+K12878+K12862+K12870+K12621+K12862+K12855+K12880+K11088
Starch and sucrose metabolism	ko00500	5	gene20412;gene9635;gene1385;gene19998;gene10969	K16055+K01194+K01196+K05349+K00693
Proteasome	ko03050	6	gene4368;gene277;gene18453;gene18519;gene19505;gene2793	K03065+K03062+K06699+K03065+K03030+K03030
Protein processing in endoplasmic reticulum	ko04141	16	gene4581;gene14191;gene2198;gene2460;gene4443;gene3049;gene20905;gene5395;gene871;gene6961;gene13298;gene7752;gene1346;gene4696;gene1200;gene13047	K08653+K14007+K16196+K09530+K07151+K14011+K14020+K10086+K09523+K04554+K13719+K03237+K04447+K09487+K07151+K14026
Peroxisome	ko04146	5	gene5710;gene12951;gene3843;gene111	K00106+K00803+K01640+K13356+K1335

			16;gene16162	6
Arginine and proline metabolism	ko00330	4	gene20083;gene9528;gene20120;gene15137	K00819+K00933+K00819+K00819
Glutathione metabolism	ko00480	5	gene9482;gene4895;gene6615;gene5089;gene3606	K11140+K10807+K11140+K11140+K01581
ABC transporters	ko02010	3	gene13649;gene3804;gene14648	K05674+K05641+K05655
RNA transport	ko03013	24	gene15859;gene4999;gene11186;gene20050;gene19176;gene18626;gene4593;gene16421;gene9120;gene3178;gene18927;gene15934;gene14023;gene9672;gene14560;gene8457;gene19042;gene12322;gene3430;gene9978;gene6835;gene3258;gene7864;gene1066	K00784+K14310+K14296+K03254+K03262+K03254+K05749+K13133+K03243+K14309+K00784+K03260+K14297+K14312+K14297+K03231+K03231+K03231+K02516+K03246+K14308+K02516+K14295+K02516
FoxO signaling pathway	ko04068	3	gene13747;gene5721;gene4697	K05868+K10305+K06631
Fatty acid metabolism	ko01212	6	gene8967;gene5274;gene11115;gene5811;gene1906;gene4995	K08766+K00665+K00645+K15013+K00665+K07509
Phagosome	ko04145	5	gene7059;gene17722;gene11286;gene7408;gene2438	K10414+K10413+K07374+K08054+K07375
Homologous recombination	ko03440	3	gene19865;gene10211;gene19446	K10875+K10875+K10873
MAPK signaling pathway - fly	ko04013	17	gene14455;gene10172;gene13213;gene9815;gene16584;gene12904;gene8764;gene13064;gene6246;gene16661;gene19399;gene16590;gene619	K05088+K20210+K20211+K20225+K13411+K08601+K17536+K08840+K14290+K05088+K20234+K20234+K20234+K07293+K13411+K08601+K0

			7;gene5001;gene12174;gene18069;gene9490	4461
Hippo signaling pathway - fly	ko04391	10	gene4601;gene2114;gene19839;gene10450;gene2634;gene16917;gene2080;gene13702;gene7569;gene14775	K06095+K16175+K16681+K16681+K16680+K16690+K05628+K16674+K16672+K16175
Glycerophospholipid metabolism	ko00564	3	gene18178;gene6748;gene68	K00111+K00623+K13511
Endocytosis	ko04144	19	gene13270;gene16896;gene12596;gene2747;gene19144;gene7709;gene11730;gene2208;gene1912;gene13361;gene4409;gene16234;gene14603;gene6481;gene5766;gene10376;gene12520;gene10994;gene13381	K19475+K12191+K04707+K12491+K18468+K11826+K10591+K12475+K12487+K11824+K04646+K05754+K01528+K05704+K07941+K06643+K11839+K11839+K05758
Aminoacyl-tRNA biosynthesis	ko00970	7	gene10866;gene9870;gene17441;gene1011;gene4066;gene13619;gene645	K14163+K01870+K14163+K01881+K01870+K01873+K01890
ECM-receptor interaction	ko04512	5	gene13844;gene17671;gene15875;gene3629;gene18353	K06254+K05637+K05635+K05719+K05637
Lysine degradation	ko00310	6	gene6663;gene21305;gene8720;gene11119;gene18804;gene19774	K09188+K11433+K09189+K14157+K11424+K06101
Phosphatidylinositol signaling system	ko04070	7	gene18679;gene16205;gene15865;gene1762;gene10872;gene15606;gene3842	K00923+K18083+K00901+K00901+K00901+K01092+K00920
Ribosome	ko03010	3	gene17400;gene7880;gene12393	K02988+K02930+K02876
Wnt signaling pathway	ko04310	9	gene7901;gene4032;gene11056;gene69;gene6039;gene14778;	K02375+K02085+K17388+K04511+K04491+K02157+K02157+

			gene14777;gene15093;gene10334	K08110+K02842
Notch signaling pathway	ko04330	5	gene18280;gene10215;gene368;gene14198;gene8074	K06052+K06052+K02599+K06057+K04505
Nucleotide excision repair	ko03420	5	gene3678;gene7471;gene9837;gene19984;gene7100	K02324+K10846+K10841+K10846+K02202
mTOR signaling pathway	ko04150	3	gene15239;gene2953;gene395	K08267+K06276+K03258
DNA replication	ko03030	4	gene7027;gene2019;gene4224;gene15139	K02212+K02321+K10742+K02320
Other types of O-glycan biosynthesis	ko00514	4	gene3691;gene539;gene7232;gene10944	K00778+K09667+K00728+K03691
Carbon metabolism	ko01200	10	gene5332;gene6689;gene7933;gene15227;gene16451;gene19172;gene20125;gene14573;gene19109;gene8062	K00164+K00036+K00164+K01895+K00140+K01900+K19269+K00605+K00844+K01053
Folate biosynthesis	ko00790	3	gene11195;gene12436;gene3017	K01930+K01077+K01077
Phototransduction - fly	ko04745	8	gene6073;gene1615;gene10149;gene13822;gene11684;gene15882;gene5881;gene489	K13805+K05692+K13803+K08834+K08834+K05692+K00910+K07972

Table S7. Predicted targets of highly expressed DEmiRNAs in *N. lugens*.

miRNA	miRNA sequence	Number of target genes	*P*-value	log_2FC	Nr annotation of major target genes
NL-miRNA-2333	UCAGCAUAGCCAGCUCUAGAUCA	11	0.01	1.41	Presenilin, Serine proteinase stubble, Sorting nexin-13-like, Bursicon, Serine/Threonine-protein kinase mTOR, ADP-ribosylation factor, Dynein beta chain
NL-miRNA-1047	CCGUGCUGGUUGGACGCCUCUCUU	24	0.02	1.22	Serine/Threonine-protein kinase PAK 3, Insulin receptor, E3 ubiquitin-protein ligase UBR2, Nuclear receptor coactivator, Neurogenic protein mastermind, Sodium channel protein para, MAP kinase-activating death domain protein, Tryptophan synthase-like, RNA polymerase II elongation factor
NL-miRNA-156	UGUCUGACAGAGAGAACAAUG	1	0.02	1.21	Thioredoxin domain-containing protein
NL-miRNA-488	UAAGGGGUGUUGGGAGGC	4	0.01	1.04	E3 ubiquitin-protein ligase, ATP-dependent DNA helicase II subunit
NL-miRNA-260	UGGCAGUGUGGUUAGCUGGUUGU	65	0.00	2.04	E3 ubiquitin protein ligase 1, Nucleoprotein TPR, Fiacylglycerol kinase theta isoform X3, Protein phosphatase PHLPP, Anaphase-promoting complex subunit, Dynein heavy chain, Tyrosine-protein kinase, Histone-lysine N-methyltransferase, NF-X1-type zinc finger protein, MAP kinase-activating death domain protein,

					Tubulin polyglutamylase, Xanthine dehydrogenase, Diacylglycerol kinase theta isoform X3, Peroxisome assembly protein, Zinc finger protein Xfin, Helicase SKI2W
NL-miRNA-1675	UUGAUGUGUGUGUGAGUG	2	0.00	1.96	LOC111061260, Cytosolic carboxypeptidase-like protein
NL-miRNA-2180	CUGGACAAGUGUGUUGAGC	1	0.02	1.58	Transient receptor potential channel pyrexia
NL-miRNA-707	CAGCCAGGUCACUCGAUAUUAUU	31	0.04	1.41	Phospholipid phosphatase, Zinc finger protein, U2 small nuclear ribonucleoprotein, Exocyst complex component, Glutamate receptor ionotropic, ly6/PLAUR domain-containing protein, Peroxisomal targeting signal 1 receptor
NL-miRNA-1385	AAGGGGUGAUGUGGAGGC	15	0.00	1.39	Glucose transporter, Probable beta-hexosaminidase, La-related protein, Proline-rich extensin-like, Disks large 1 tumor suppressor protein, Sine oculis-binding protein homolog, Serine/Threonine-protein kinase
NL-miRNA-1547	CGGUUGAUAACUGCAGUCUUGGGGC	97	0.02	1.38	DNA topoisomerase, Sodium-dependent multivitamin transporter, Poly(U)-specific endoribonuclease homolog, DNA (cytosine-5)-methyltransferase, Serine/Arginine repetitive matrix protein, Deoxyribose-phosphate aldolase, E3 ubiquitin-protein ligase, Programmed cell death protein, Peroxidase, Neuropilin and tolloid

NL-miRNA-1982	UCAUCCUCCUCCUCUUCAUC	60	0.00	2.42	Serine/Threonine-protein kinase, Histone acetyltransferase, E3 ubiquitin-protein ligase and extracellular sulfatase, Methyltransferase-like protein, Acidic leucine-rich nuclear phosphoprotein, ATP-binding cassette sub, Zinc finger protein, Adenylate kinase, Suppressor protein, Histone acetyltransferase, Phosphatase and actin regulator
NL-miRNA-1147	GGAGUGAUCCGUGGUCUAGUGGAU	81	0.00	2.12	Putative mediator of RNA polymerase II, GTPase-activating protein, Phenoloxidase, Odorant receptor, Flocculation protein, Homeobox protein HMX3-like, Rac GTPase-activating protein, Protein drumstick isoform X2
NL-miRNA-453	UAUCACAGCCGUAAUUCCUGUG	1	0.00	2.08	Phospholipid-transporting ATPase
NL-miRNA-1462	GGAGUGAUCCGUGGUCUAGUGGAU	81	0.00	2.08	Serine/threonine-protein kinase, GTPase-activating protein, Histone acetyltransferase KAT6A isoform X1, Odorant receptor, DNA-directed RNA polymerase, E3 ubiquitin-protein ligase, Fatty acid synthase
NL-miRNA-54	AGACAACGGUCCGCUGUGCCCUGGG	41	0.00	2.05	Heparan sulfate glucosamine, tRNA modification GTPase, Histone-lysine N-methyltransferase, Glucose-6-phosphate 1-dehydrogenase, Hcylglycerol kinase, Pyrethroid hydrolase Ces2a-like

NL-miRNA-1546	UGGAACUGGUUCUGGAAC	6	0.00	1.40	Transcriptional regulator, Histone acetyltransferase, GATA zinc finger domain-containing protein, Extensin-like isoform X1
NL-miRNA-756	GUCACGGAGGUGGUCACGGA	29	0.01	1.33	Activating signal cointegrator, Tripeptidyl-peptidase, Pyruvate carboxylase, Transmembrane protein, Ubiquitin-protein ligase E3C, Dynein heavy chain
